# Pharmacophore-Based Screening, Molecular Docking, and Dynamic Simulation of Fungal Metabolites as Inhibitors of Multi-Targets in Neurodegenerative Disorders

**DOI:** 10.3390/biom13111613

**Published:** 2023-11-04

**Authors:** Danish Iqbal, Mohammed Alsaweed, Qazi Mohammad Sajid Jamal, Mohammad Rehan Asad, Syed Mohd Danish Rizvi, Moattar Raza Rizvi, Hind Muteb Albadrani, Munerah Hamed, Sadaf Jahan, Hadeel Alyenbaawi

**Affiliations:** 1 Department of Health Information Management, College of Applied Medical Sciences, Buraydah Private Colleges, Buraydah 51418, Saudi Arabia; 2 Department of Medical Laboratory Sciences, College of Applied Medical Sciences, Majmaah University, Majmaah 11952, Saudi Arabia; m.alsaweed@mu.edu.sa (M.A.); s.jahan@mu.edu.sa (S.J.); 3 Department of Health Informatics, College of Public Health and Health Informatics, Qassim University, Al Bukayriyah 52741, Saudi Arabia; m.quazi@qu.edu.sa; 4 Department of Basic Medical Science, College of Medicine, Majmaah University, Al Majmaah 11952, Saudi Arabia; mr.asad@mu.edu.sa; 5 Department of Pharmaceutics, College of Pharmacy, University of Ha’il, Ha’il 81442, Saudi Arabia; sm.danish@uoh.edu.sa; 6 School of Allied Health Sciences, Manav Rachna International Institute of Research & Studies (MRIIRS), Faridabad 121001, India; rajrizvi@gmail.com; 7 Department of Clinical Laboratory Sciences, College of Applied Medical Sciences, Imam Abdulrahman Bin Faisal University, Dammam 34212, Saudi Arabia; hmalbadrani@iau.edu.sa; 8 Department of Pathology, Faculty of Medicine, Umm Al-Qura University, Makkah 21955, Saudi Arabia; mhhamed@uqu.edu.sa

**Keywords:** Alzheimer’s disease, fungal metabolites, GSK-3β, NMDA receptor, BACE-1, multitarget inhibitor, molecular dynamics simulation, pharmacophore modeling

## Abstract

Neurodegenerative disorders, such as Alzheimer’s disease (AD), negatively affect the economic and psychological system. For AD, there is still a lack of disease-altering treatments and promising cures due to its complex pathophysiology. In this study, we computationally screened the natural database of fungal metabolites against three known therapeutic target proteins of AD. Initially, a pharmacophore-based, drug-likeness category was employed for screening, and it filtered the 14 (**A**–**N**) best hits out of 17,544 fungal metabolites. The 14 best hits were docked individually against GSK-3β, the NMDA receptor, and BACE-1 to investigate the potential of finding a multitarget inhibitor. We found that compounds **B**, **F**, and **L** were immuno-toxic, whereas **E**, **H**, **I**, and **J** had a higher LD_50_ dose (5000 mg/kg). Among the examined metabolites, the Bisacremine-C (compound **I**) was found to be the most active molecule against GSK-3β (ΔG: −8.7 ± 0.2 Kcal/mol, Ki: 2.4 × 10^6^ M^−1^), NMDA (ΔG: −9.5 ± 0.1 Kcal/mol, Ki: 9.2 × 10^6^ M^−1^), and BACE-1 (ΔG: −9.1 ± 0.2 Kcal/mol, Ki: 4.7 × 10^6^ M^−1^). It showed a 25-fold higher affinity with GSK-3β, 6.3-fold higher affinity with NMDA, and 9.04-fold higher affinity with BACE-1 than their native ligands, respectively. Molecular dynamic simulation parameters, such as RMSD, RMSF, Rg, and SASA, all confirmed that the overall structures of the targeted enzymes did not change significantly after binding with Bisacremine-C, and the ligand remained inside the binding cavity in a stable conformation for most of the simulation time. The most significant hydrophobic contacts for the GSK-3β-Bisacremine-C complex are with ILE62, VAL70, ALA83, and LEU188, whereas GLN185 is significant for H-bonds. In terms of hydrophobic contacts, TYR184 and PHE246 are the most important, while SER180 is vital for H-bonds in NMDA-Bisacremine-C. THR232 is the most crucial for H-bonds in BACE-1-Bisacremine-C and ILE110-produced hydrophobic contacts. This study laid a foundation for further experimental validation and clinical trials regarding the biopotency of Bisacremine-C.

## 1. Introduction

Neurodegenerative diseases are incurable and devastating disorders that have great impacts on a person’s psychological, mental, physical, and financial states. Neurological illnesses afflict almost 1 billion individuals globally; of these individuals, 50 million have epilepsy, and 24 million have Alzheimer’s disease and other types of dementia. Alzheimer’s disease (AD) is the fifth most prominent reason for mortality globally, and it is ranked sixth in Saudi Arabia (currently more than 130,000 cases) [1,2]. AD patients exhibit memory loss, cognitive impairments, problems related to speaking or writing, the inability to practice self-care, aberrant motor behavior, agitation, apathy, and dysphoria [3]. Due to the complex pathology of AD, its exact mechanism is not fully understood. However, a large quantity of evidence has extensively highlighted the role of the amyloidogenic pathway: the hyper-phosphorylation and aggregation of tau, the alteration of the cholinergic pathway, and oxidative stress in the pathophysiology and progression of AD [3,4]. The aberrations in these pathways involve changes in some neurotransmitters, neurotrophins, receptors, enzymes, and gene expressions, such as hyperactivation of the NMDA receptor (N-methyl-D-aspartate receptor), monoamine oxidases, beta-secretase (BACE-1), GSK-3β (glycogen synthase kinase), and AChE (acetylcholinesterase) [5,6,7,8].

To date, there is still a lack of disorder-altering treatments and cures for AD. There are only two classes of FDA-approved medication available that are used for the management of AD, both of which are protein (enzyme or receptor) inhibitors [9]. However, there are various enzymes and receptors involved in the pathogenesis and progression of AD, including BACE-1, GSK-3β, CDK5, AChE, BuChE, MAO-A, MOA-B, NMDA, and ROCK2, that can be targeted therapeutically—either individually or collectively [10,11,12,13,14,15]. 

In AD patients, hyperactivation of GSK-3β, the NMDA receptor (NMDA), and BACE-1 have been shown to be involved in the development and deposition of one or more of the main pathological hallmarks of AD, and specifically, the formation of amyloid plaques and neurofibrillary tangles. Consequently, these alterations can lead to impairment of neurotransmission and neurodegeneration [16]. β-secretase (BACE-1) is one of the essential enzymes for the cleavage of the β-amyloid precursor protein (APP) into the amyloid beta peptides 42 (Aβ-42), which are prone to aggregate and eventually form amyloid plaques. On the other hand, another study showed that the accumulation of amyloid plaques and reactive oxygen species may cause GSK-3β to become hyperactive, which enhances tau protein phosphorylation and facilitates the development of neurofibrillary tangles [17]. Additionally, neuronal death and excitotoxicity result from the overactivation of NMDA in conjunction with nerve fiber tangles and amyloid plaques [5,18].

Several clinical trials are ongoing regarding the development of novel drugs for use against pathogenesis and for the treatment of AD by inhibiting various targets and inducing several genes and proteins, individually or in combination, as a multitarget approach [19,20]. Multitarget-directed ligands (MTDLs) are thought to be a more effective treatment option than the single-target approach for the management of Alzheimer’s disease due to the complex nature of the disease, where several pathways and a variety of proteins are involved in its development [11,12,13,21,22]. Enzyme/protein inhibition using several in silico approaches is the initial choice for screening novel inhibitors for many drug developers [11,12,23]. Thanks to recent advancements in computing capacity, it has become possible to develop several cheminformatic approaches in order to obtain rapid screening and optimization of chemical entities [24,25,26,27]. 

Natural products have been shown to have a wide range of therapeutic actions that control various negative pathophysiological consequences of oxidative damage, including bacterial infection, ulceration of the stomach, elevated cholesterol levels, carcinoma, and neurological ailments [13,28,29,30,31]. In earlier studies, the therapeutic applications and mode of action of polyphenols (including curcumin, piperine, and resveratrol) toward age-associated neurological diseases were explained. In this explanation, the significance of the olive polyphenol modulation of Nrf2-related stress-responsive genes, which, in accordance with the hormesis theory, activate this neuroprotective cascade to maintain brain health, was highlighted, as well as offering potential application in the prevention and treatment of aging and age-related cognitive disorders in people [32,33]. Moreover, some other natural compounds, such as geraniol, soyasapogenol-B, taurine, and nobiletin, showed promising results for the management of neurological manifestations [13,30,34,35]. Similar to natural products, works have shown the therapeutic potential of the use of fungal metabolites against various disorders [36,37]. For example, the extracellular material of the fungus *Fusarium oxysporum* has been utilized for the preparation of nanoparticles and found to be an effective agent for the management of pathogenic microbes [38]. Butyl xanalterate is another fungal metabolite reported as a prominent CK-2α inhibitor that could become a cancer (chronic lymphocytic leukemia) management drug [31]. 

Therefore, we aim to investigate new treatments derived from fungal metabolites using molecular docking and ADMET methods, as well as to examine their therapeutic multitarget potential against three important AD target proteins (GSK-3β, NMDA receptor, and BACE-1). Additionally, we plan to use molecular dynamics simulation (MDS) to evaluate the best hits obtained. To the best of our knowledge, this chemical library has never been investigated for use in treating AD by targeting the GSK-3β receptor, NMDA receptor, and BACE-1.

## 2. Material and Methodologies

### 2.1. Tools Used for Computational Study

The online available PDB database (http://www.rcsb.org/pdb/), accessed on 15 October 2021 [39], was used to download the 3D co-crystallized protein structures and 3D-conformers of fungal metabolites were retrieved from the PubChem database [40]. Lamarkian genetics was used as a scoring function. AutoDock-based PyRx-Python 0.8 tool was used for molecular docking [41,42]. To visualize the molecular interactions, we used the Biovia Discovery Studio visualizer (2020, Dassault Systèmes, 175 Wyman Street, Waltham, MA, USA) [43]. Molecular dynamics simulation was carried out using Desmond (Shchrodinger-2020, LLC, New York, NY, USA) [44,45]. All the computational analysis was carried out on an Intel Xenon workstation (E3-1245-8C) with a 3.50 GHz processor, 28 GB of RAM, and NVIDIA Quadro P5000 GPU served as a graphic card. 

### 2.2. Preparation of Ligands

The fungal metabolites (17,544 compounds) were retrieved from the PubChem database (accessed on 25 October 2021) and processed to filter out the metabolites based on drug-likeness properties, most importantly, the blood–brain barrier-positive. The filtered fungal metabolites were further used for pharmacophore-based virtual screening. After that, molecular docking checks were carried out to establish the inhibitory potential of best hits [46]. The “.sdf” file of finalized (filtered and screened) ligands (3D conformers) was downloaded from the PubChem database (https://pubchem.ncbi.nlm.nih.gov/), accessed on 25 October 2021. These ligands were then energy minimized using the universal force field (UFF). Then, they were converted to AutoDock-suitable “.pdbqt” file format through the inbuilt OpenBabel tool in PyRx software (PyRx-Python 0.8 tool). 

### 2.3. Preparation of Target Proteins

The 3D coordinates of the three target proteins, namely glycogen synthase kinase-3beta (GSK-3β), beta-secretase (BACE-1), and N-methyl-D-aspartate, receptor glycine binding sites (NMDA) were downloaded with PDB id: 1J1C [47], 1W51 [48], and 1PBQ [49], respectively, from the PDB database. The binding pockets of individual target proteins were selected on the basis of previous reports. The target proteins were prepared by removing the ligand and water molecules, assigning hydrogen polarities, computing Gasteiger charges, and converting the structure from .pdb to. pdbqt file format. The preparation of the structure in “.pdbqt” file format was finalized for computational investigation. The use of a built-in tool (OpenBabel) in PyRx, energy minimization, and optimization of all structures was carried out.

### 2.4. Pharmacophore Generation and Virtual Screening

Ligand-based pharmacophores were generated using the MOE server [50]. The three co-crystallized native ligands of their respective proteins (PDB ID: 1J1C, 1PBQ, and 1W51) were used to generate the query based on pharmacophore features. The chosen compounds were matched for a broad spectrum of structural diversity as well as activity. To combine all the characteristics of the chosen drugs, a pharmacophore model was created. A sizable set of hydrogen bond donors (Don), hydrogen bond acceptors (Acc), aromatic centers (Aro), donors and acceptors (Don & Acc), and other pharmacophore properties were included in this list. Based on drug-similarity features, filtered fungal metabolites were employed to identify the active pharmacophore across the compounds in the library. Molecules with low RMSD (≤0.9 Å) values from the pharmacophore active sites were screened and filtered out of the database’s numerous hit compounds to conduct docking investigations [51,52,53]. 

### 2.5. Molecular Docking

Utilizing the Lamarckian genetic algorithm approach, molecular docking was carried out, employing the PyRx-Python 0.8 screening program paired with AutoDock 4.2 [41,42]. Each of the targeted enzymes was docked with each of the ligands separately during different docking sessions. The binding pockets were used as similar coordinates of active sites for co-crystallized native ligands with their respective proteins (PDB ID: 1J1C, 1PBQ, and 1W51). The binding pockets were determined by the position of active site residues for 1J1C (GLY65, VAL70, ALA83, LYS85, ASP133, TYR134, VAL135, GLN185, LEU188, ARG141), 1PBQ (PHE16, PHE92, PRO124, THR126, ARG131, TRP223, ASP224, VAL227, PHE250), and 1W51 (TYR71, THR72, GLN73, ILE126, ASP228, GLY230, THR232). The grid dimensions for the target proteins were set to 25 x 25 x 25 Å^3^ and centered at x: 20.30; y: 16.49; z: −10.38 for GSK-3β (PDB id: 1J1C), x: 5.64; y: 37.82; z: −17.06 for NMDA (PDB id: 1PBQ), and x: 68.73; y: 47.61; z: 7.69 for BACE-1 (PDB id: 1W51), respectively. The “exhaustiveness” setting for the docking was set to 8. The software’s default values were used for all other docking parameters. The following relationship was used to compute the binding affinity (Ki) of ligands for the target enzyme from the binding energy (ΔG) [11,12]:

ΔG = −RT lnKd(1)

where R and T, respectively, stood for universal gas constant and temperature. The ligands with the lowest binding energies were chosen for additional examination. Using Discovery Studio 2020 (BIOVIA), the ideal posture of each “protein–ligand complex” was developed and examined.

### 2.6. Prediction of Physicochemical Properties and Toxicity Level

The web-based tools SwissADME (http://www.swissadme.ch), accessed on 5 May 2022 [54], and ProTox-II [55] were used to establish the physicochemical properties and toxicity potentials, respectively, of the best fungal metabolite hits after pharmacophore modeling. 

### 2.7. Molecular Dynamics (MD) Simulation

Using “Desmond (Schrodinger-2020, LLC, New York, NY, USA)”, the best-scoring ligand’s MD simulation was carried out in complex with each of its targeted enzymes (GSK-3β, NMDA, and BACE-1), in triplicate as previously mentioned [12,44]. After molecular docking, the best-hit protein–ligand complexes were loaded into Schrodinger software’s Maestro interface. Complicated optimization and reduction techniques were employed to preprocess the protein–ligand complexes using the Maestro tool’s protein preparation wizard. The System Builder tool was used to prepare each system. A solvent model named TIP3P, with an orthorhombic box (10 Å), was used. The OPLS 2005 force field was employed in the simulation process [56]. The models were neutralized by the addition of counterions. The addition of 0.15 M sodium chloride (NaCl) simulated physiological circumstances. For the duration of the simulation, the NPT ensemble with a 300 K temperature and 1 atm pressure was used. Before the simulation, the models were relaxed. After every 100 ps, the trajectories were kept for analysis. To determine the stability of protein–ligand complexes, variables like RMSD (root-mean-square deviation), RMSF (root-mean-square fluctuation), Rg (radius of gyration), SASA (solvent-accessible surface area), SSE (secondary structure elements), and interactions of protein and ligand were examined. The findings of the three separate studies are shown as mean ± standard deviation [57,58].

### 2.8. Calculations of Free Energy (Prime-MM/GBSA)

Using Prime Module (Schrodinger, LLC, New York, USA), the binding free energies of each protein–ligand complex were calculated using the MM-GBSA method, as previously mentioned [11,12]. This method involved computing free energy on the last 10 ns of the MD simulation trajectories after the equilibrium was established. Concisely, the docked complexes underwent local optimization using molecular mechanics (MM) for Prime initially, and then their energies were reduced using the OPLS-AA (2005) force field and the generalized Born surface area (GBSA) continuum solvent framework. The formula for calculating the binding free energy (ΔGBind) is:

ΔGBind = ΔGCoulomb + ΔGvdW + ΔGCovalent + ΔGH−bond + ΔG Sol_Lipo + ΔGSolv_GB + ΔGPacking + ΔGSelf−contact

## 3. Results and Discussion

### 3.1. Criteria for Selecting Compounds during Retrieval

**Figure 1:**
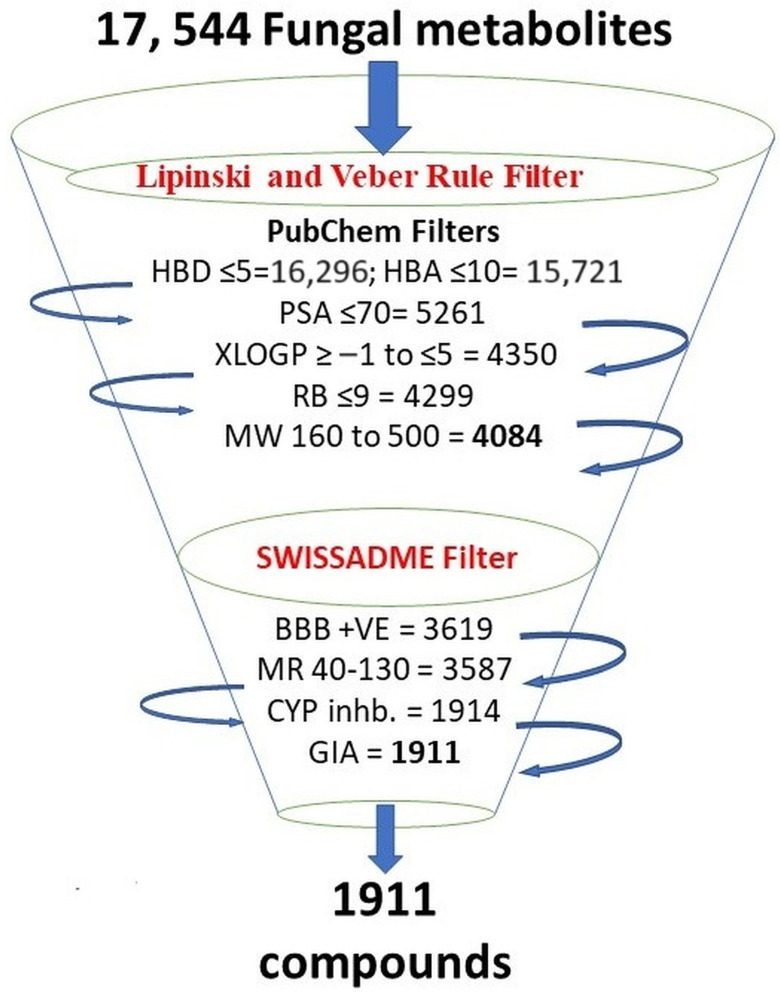
Selection criteria of fungal metabolites. Initial compounds retrieved, and filtered compounds by following different filters represented in bold. Tools and filters used in the study are shown in red.

We filtered the 17,544 fungal metabolites available in the natural product atlas of the PubChem database based on their drug-likeness criteria (assessed on 25 October 2021). These parameters include molecular weight (160 to 500 g/mol), rotatable bond (0 to 9), polar surface area (≤70 Å²), hydrogen bond donor (0 to 5), hydrogen bond acceptor (0 to 10), and XLogP (−1 to 5). These criteria allowed us to narrow down the number to 4084 metabolites. After that, we used the SWISSADME tool to filter out 1911 metabolites for further study. These metabolites showed high GI (gastrointestinal) absorption, molar refractivity (40 to 130), blood–brain barrier-positive, and no cytochrome P450 (CYP) inhibitors (Figure 1). The reason the majority of medications failed during clinical trials and the drug development process is now widely recognized and discussed in several papers is that drug-like substances have to have specific criteria called Lipinski’s rules [59,60]. The four physicochemical parameters, namely molecular weight (MW) between 160 and 500 g/mol, XlogP between −1 and 5, hydrogen bond donors (HBD) less than 5, and hydrogen bond acceptors (HBA) less than 10, have been represented by 90% of orally active drugs that have completed clinical phase-2 trial. The oral bioavailability of compounds with more than 10 rotatable bonds is typically low [61]. For improved intestinal and oral absorption, the molar refractivity (MR) range is thought to be between 40 and 130 [62]. Those substances that adhere to at least three of the Lipinski rule’s five criteria can be considered drug-like in nature [60]. Moreover, a Polar surface area (PSA) of less than 70 Å^2^ represents good absorption and distribution and can easily be permeable to the blood–brain barrier, which is an important criterion for developing oral medications against neurodegenerative disorders [63,64]. Drugs that have a therapeutic role for any other disease except central nervous system disorder should not be permeable to the blood–brain barrier [59].

### 3.2. Pharmacophore Modeling and Screening of Compounds

**Figure 2:**
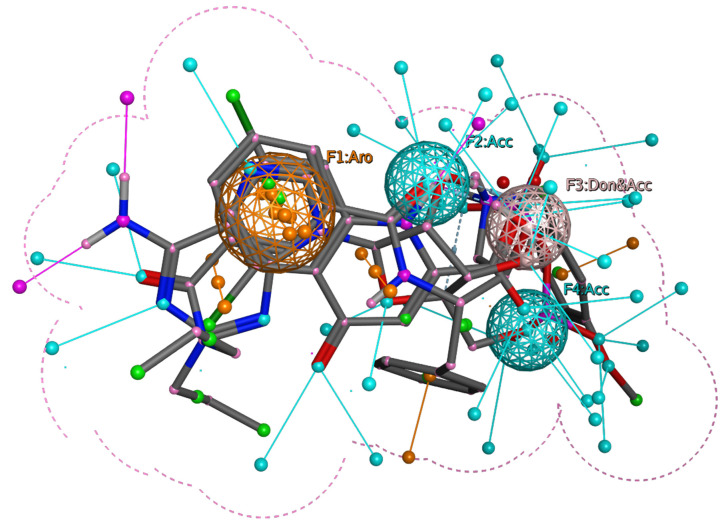
The pharmacophore hypothesis is based on three aligned ligands. The brown spheres represent aromatic features (F1:Aro), the cyan spheres represent H-bond acceptors (F2 and F4:Acc), and the pink spheres represent mixed H-bond donors and acceptors (F3: Don & Acc).

We used three native ligands co-crystallized with their respective proteins (GSK-3β, NMDA, and BACE-1) to generate the query based on pharmacophore features. As shown in Figure 2, the brown spheres represent aromatic features (F1:Aro), the cyan spheres represent H-bond acceptors (F2 and F4:Acc), and the pink spheres represent mixed H-bond donors and acceptors (F3: Don & Acc). We screened all 1911 fungal metabolites and finalized the 14 best hits (3A-3N) that have RMSD less than 0.9 Å and with similar or compliance with the crucial pharmacophore features of the native ligands of three different proteins, respectively (Figure 2).

**Figure 3:**
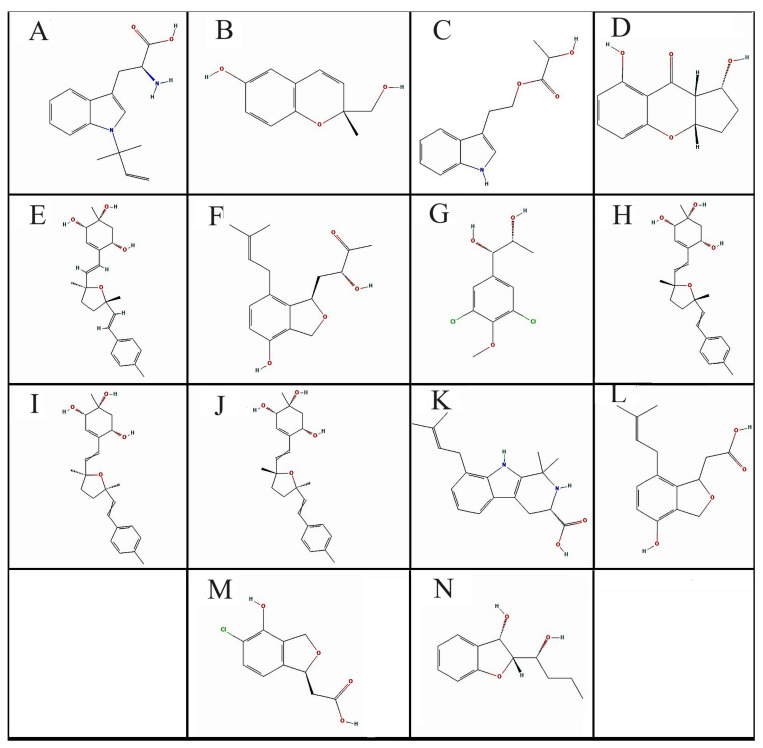
2D structure of the selected compounds (**A**–**N**).

The structural information of these 14 compounds showed that four of the compounds (**E**, **H**, **I**, and **J**) are isomers of Bisacremine, which only differentiate in the two-methyl group in the tetrahydrofuran ring, which is in a cis-configuration similar to the configuration of the hydroxyl group (Figure 3).

### 3.3. Physicochemical and Pharmacokinetics Parameters

The physicochemical properties of the top 14 best hits are shown in Table 1. All 14 compounds’ molecular weights are in the range of 192.21 to 384.51, the number of hetero atoms is 14 to 28, aromatic heteroatoms range from 6 to 9, F-Csp3 ranges from 0.27 to 0.5, the number of rotatable bonds from 0 to 5, the number of hydrogen bond acceptors from 3 to 4, the number of hydrogen bond donors from 2 to 3, molar refractivity from 53.75 to 113.11, TPSA from 49.69 to 69.92 Å^2^, and XlogP3 from 0 to 2.36. Higher XlogP values are more soluble in non-polar solvents, and lower XlogP values are more soluble in water. We observe that **E** and **H**–**J** have the same values for all the selected parameters. Therefore, they may be isomers. 

### 3.4. Toxicity Prediction

Furthermore, we also evaluated the toxicity profile and predicted LD_50_ for all these 14 compounds, and from our results (Table 2), it has been illustrated that most of the compounds are non-toxic (IA) for all the toxicity parameters with high probability where few compounds, such as **B**, **F**, and **L**, were found to be immuno-toxic with 0.75, 0.83, and 0.69 probability, respectively. Moreover, the predicted LD_50_ (mg/kg B.W.) values showed that **E**, **H**, **I**, and **J** have higher LD_50_ doses (5000 mg/kg), followed by **C** (3500 mg/kg), and the rest of the compounds were found to have ≤1500 mg/kg of LD_50_ dose. A higher value for LD_50_ is considered to be safe for medicinal drugs.

### 3.5. Molecular Docking and Interactions Analysis

It is widely acknowledged that the computational screening of a large number of small organic compounds for their antagonistic potential towards the target proteins may significantly decrease the time, expense, and effort of wet-lab high-throughput screening [26,27]. In this study, 3D conformers (117) of the 14 best metabolite hits from pharmacophore-based screening were retrieved from the PubChem database and individually docked with the target proteins (GSK-3β, NMDA, and BACE-1) to analyze the binding energy (ΔG) and binding affinity (Ki). 

The molecular docking methodology was initially verified by redocking the native ligands in the corresponding proteins, and it was discovered that it binds to almost identical residues. Between docked and native ligands, the RMSD value (≤2 Å) was within an acceptable range. Adenosine-5′-Diphosphate, 5,7-Dichlorokynurenic acid, and a non-peptidic inhibitor were the native ligands of the NMDA receptor, GSK-3β, and BACE-1, respectively, and were used as reference ligands for the 117 conformer molecules of fungal metabolites. 

The findings showed that only five compounds—out of a total of 14—exhibited stronger binding affinities with all three targets compared to reference ligands and were therefore deemed multitarget inhibitors (Table 3). Interestingly, we noticed that among these five compounds, four are the isomers of Bisacremine, where Bisacremine-C (**I**) was found to be most active molecule against GSK-3β (ΔG: −8.7 ± 0.2 Kcal/mol, Ki: 2.4 × 10^6^ M^−1^), NMDA (ΔG: −9.5 ± 0.1 Kcal/mol, Ki: 9.2 × 10^6^ M^−1^), and BACE-1 (ΔG: −9.1 ± 0.2 Kcal/mol, Ki: 4.7 × 10^6^ M^−1^). The compound **I** (Bisacremine-C) is the best hit in which the two-methyl groups in the tetrahydrofuran ring are in cis-configuration similar to the configuration of the hydroxyl group. Hence, fewer steric hindrances were observed, and therefore, it has better binding interactions with target proteins. The compound Bisacremine-C has most of the structural similarity with the native ligands of selected proteins, which has been highlighted in the supplementary file (Supplementary Figure S1). Bisacremine-C (3I) is a dimeric acremine that was initially isolated from the *Acremonium persicinum* strain by Wu et al. [65], and they explored the bioactive potentials of this compound against several cell lines (HeLa—derived from cervical cancer cells; A549—adenocarcinomic human alveolar basal epithelial cells; and HepG2 cells—human liver cancer cell line).

#### 3.5.1. Molecular Interaction Analysis of Glycogen Synthase Kinase 3 Beta (GSK-3β) and Best-Hit Ligand

**Figure 4:**
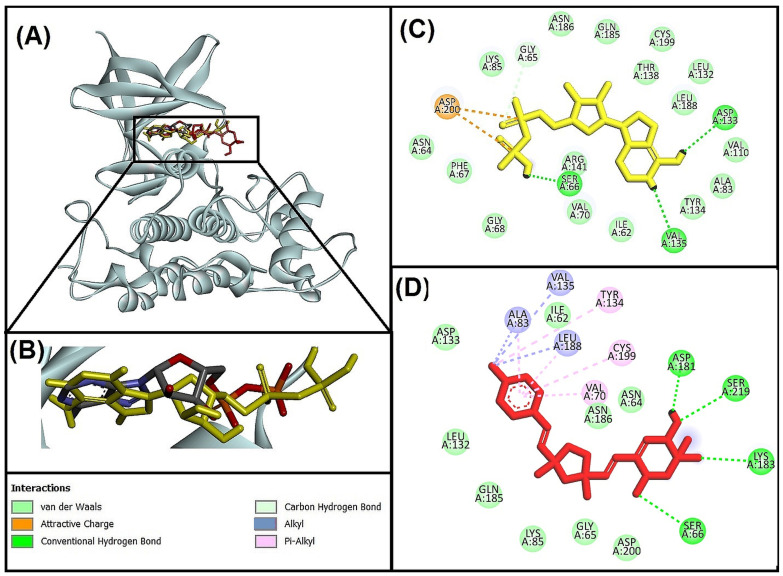
(**A**) Superimpose image of docked ligands in the active site of GSK-3β (PDB I’d: 1J1C) (native ligand: gray; Redocked native ligand: yellow; Bisacremine-C (Compound I’d-139587420): Red in the catalytic active site (**B**) Superimpose zoom in the image of native ligand and redocked, Molecular interactions analysis of (**C**) redocked ligand and (**D**) best-docked ligand (Bisacremine-C) with GSK-3β (1J1C) enzyme.

In this study, we investigated the molecular interaction between compound **I** (Bisacremine-C) and GSK-3β through Discovery Studio visualizer tools. We observed that the best-hit ligand (Bisacremine-C) and native ligand as reference inhibitor (Adenosine-5’-Diphosphate) occupied a similar catalytic site in the target protein (GSK-3β) as shown in Figure 4A,B, where the reference inhibitor and GSK-3β complex has been stabilized by two electrostatic attractive charges between LIG:P-ASP200:OD2, five conventional hydrogen bonds between LIG:HN-VAL135:O, LIG:H-ASP133:O, LIG:H-LIG:O, LIG:H-SER66:OG, LIG:H-LIG:O, and one carbon–hydrogen bond between GLY65:CA-LIG:O. Moreover, several active site residues participate in forming van der Walls interactions to stabilize the complex (Figure 4C). Although the Bisacremine-C (best hit) and GSK-3β complex has been stabilized with five conventional hydrogen bonds between SER66:N-LIG:O, LYS183:NZ-LIG:O, SER219:OG-LIG:O, LIG:H-ASP181:OD2, three alkyl hydrophobic interactions between ALA83-LIG:C, LIG:C-VAL135, LIG:C-LEU188, five Pi-alkyl hydrophobic interactions between TYR134-LIG:C, LIG-VAL70, LIG-ALA83, LIG-LEU188, LIG-CYS199. Moreover, several active site residues are involved in making van der Walls interactions between the complex (Figure 4D). We also noticed that the compound Bisacremine-C bound with a 25-fold higher affinity with GSK-3β than the native ligand.

Earlier in vitro research revealed that GSK-3β is capable of modulating presenilin-1 function to control the generation of pathogenic Aβ42 oligomers. In vitro and transgenic AD animal-model studies have demonstrated that Aβ42 promotes GSK-3β signaling; furthermore, GSK-3β activity increased significantly in AD patient brains. The hyperactivation of GSK-3β is associated with the abnormal phosphorylation of tau proteins in AD, leading to the development of neurofibrillary tangles. GSK-3β inhibition, however, lessens BACE-1-mediated APP breakdown using an NF-kB signaling-mediated strategy. The outcome suggests that blocking GSK-3β reduces the illness linked to Aβ pathology [15]. 

Key residues (VAL135 and ASP133) are accessible in the ATP-binding site, sometimes referred to as the activation loop, of the GSK-3β protein, which has two active sites: ATP-binding and substrate-binding sites, LYS85 and GLU97, additionally have a significant part in the catalytic procedure [66]. According to a prior study, ARG141 is one of the crucial residues for TPK I/GSK-3β to recognize ATP/ADP specifically. Other crucial residues in ATP-binding sites include ILE62, VAL70, ALA83, LYS85, VAL110, LEU132, GLN185, LEU188, and ASP200 [47]. Our findings show that most of the Important residues (ILE62, ASN64, GLY65, SER66, VAL70, ALA83, LYS85, LEU132, ASP133, TYR134, VAL135, GLN185, ASN186, and LEU188) were often interacted with by both the reference ligand (Adenosine-5′-Diphosphate) and Bisacremine-C compound. Our results correspond with previously published reports [66,67].

#### 3.5.2. Molecular Interaction Analysis of N-methyl-D-Aspartate Receptor (NMDA) and Best-Hit Ligand

**Figure 5:**
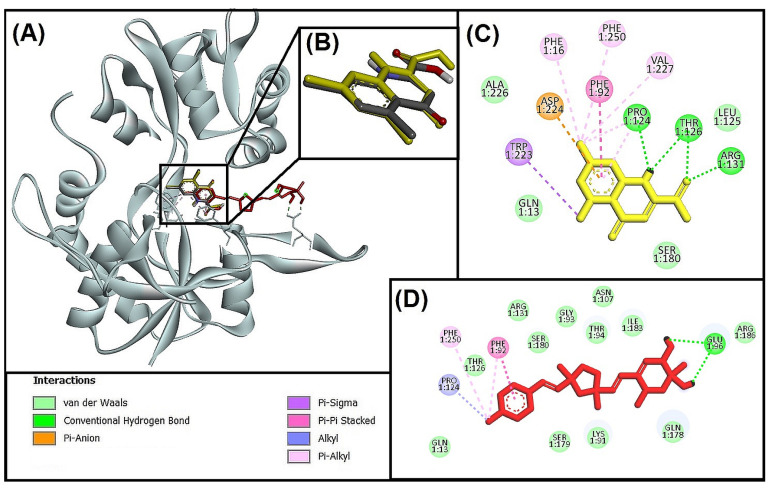
(**A**) Superimposed image of docked ligands in the active site of NMDA (PDB I’d: 1PBQ) (native ligand: Gray; Redocked native ligand: Yellow; Bisacremine-C (Compound I’d-139587420): Red in the catalytic active site (**B**) Superimpose zoom of the image of native ligand and redocked, molecular interactions analysis of (**C**) redocked ligand, and (**D**) best-docked ligand (Bisacremine-C) with NMDA (1PBQ) enzyme.

We investigated the molecular interaction between compound **I** (Bisacremine-C) and NMDA through Discovery Studio visualizer tools. We observed that the best-hit ligand (Bisacremine-C) and native ligand as reference inhibitor (5,7-Dichlorokynurenic acid) occupied a similar catalytic site in the target protein (NMDA) as shown in Figure 5A,B, where reference inhibitor and NMDA complex has been stabilized by four conventional hydrogen bonds between the THR126:HN-LIG:O, ARG131:HH12-LIG:O, LIG:HN-PRO124:O, LIG:HN-THR126:OG1, one Pi-anion electrostatic interaction between ASP224:OD2–LIG, seven hydrophobic interactions between LIG:Cl-TRP223 (Pi-sigma), PHE92-LIG (Pi-Pi stacked), LIG:Cl-PRO124 (alkyl), LIG:Cl-VAL227 (alkyl), PHE16-LIG:Cl (Pi-alkyl), PHE250-LIG:Cl (Pi-alkyl), LIG-PRO124 (Pi-alkyl). Moreover, several active site residues participate in forming van der Walls interactions to stabilize the complex (Figure 5C). However, the Bisacremine-C (best hit) and NMDA complex have been stabilized with two conventional hydrogen bonds between LIG:H-GLU96:OE1, and LIG:H-GLU96:OE2, one Pi-Pi stacked hydrophobic interaction between PHE92–LIG, one alkyl hydrophobic interaction between LIG:C-PRO124, and two Pi-alkyl hydrophobic interactions between PHE92-LIG:C and PHE250-LIG:C. Moreover, several active site residues are involved in making van der Walls interactions between the complex (Figure 5D). We also noticed that the compound Bisacremine-C binds with a 6.3-fold higher affinity with NMDA than the native ligand.

NMDA receptor signaling at synapses is essential for neuronal survival. A major factor in reversing the synaptic pro-survival signaling pathway and tilting the scales in favor of excitotoxicity and ultimate neurodegeneration is the overproduction of glutamate by astrocytes or presynaptic terminals. In individuals with moderate to severe AD, memantine, an FDA-approved NMDA receptor inhibitor, has demonstrated beneficial therapeutic effects. By decreasing extra-synaptic NMDA receptor signaling, it could do this. Consequently, it is advantageous to focus on NMDA receptors for the treatment of AD [68].

Glycine and glutamate are both necessary for the activation of NMDA receptors, with NR1 and NR2 constituting the corresponding glycine and glutamate sites. The antagonist 5,7-dichloro kynurenic acid (DCKA) co-crystallized high-resolution structure (1.90 Å) of NR1 ligand-binding core was used in this study. The therapeutic potential of the NR1 site has been considered [49,69]. The amino acid residues PRO124, THR126, and ARG131 are crucial for blocking the Gly/NMDA receptor, according to Ugale and Bari [25,70]. With some additional interactions (GLN13, TRP223, and ASP224), Devid et al. [71] also noted these interactions. Our findings are consistent with these earlier publications.

#### 3.5.3. Molecular Interaction Analysis of Human Beta-Secretase (BACE-1) and Best Hit Ligand

**Figure 6:**
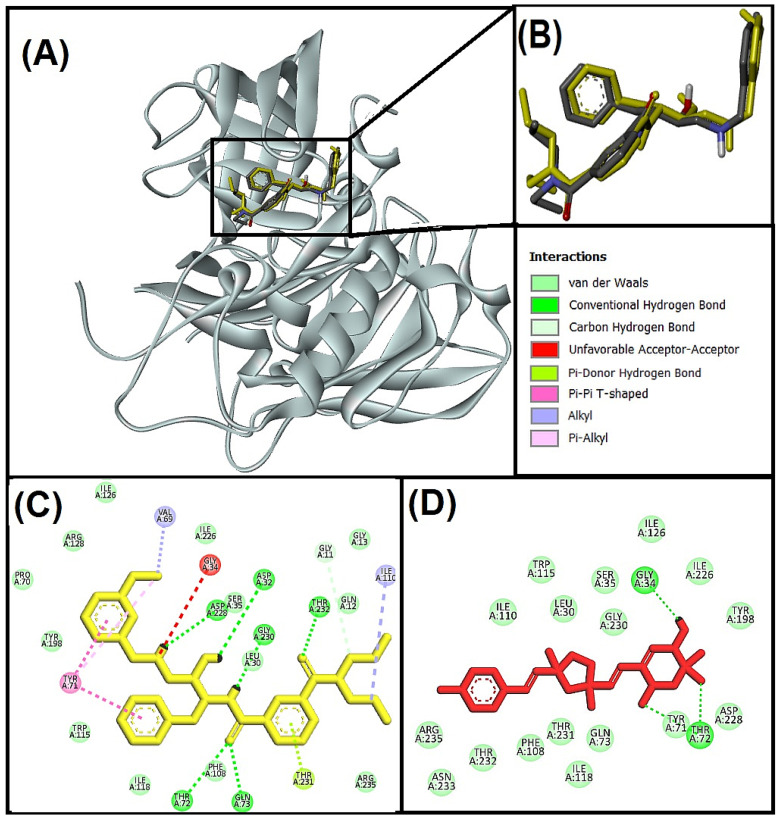
(**A**) Superimposed image of BACE-1 active site docked with ligands (PDB I’d: 1W51) (native ligand: gray; Redocked native ligand: yellow; Bisacremine-C (Compound I’d-139587420): red in the catalytic active site (**B**) Superimposed zoom in the image of native ligand and redocked, Molecular interactions analysis of (**C**) redocked ligand and (**D**) best-docked ligand (Bisacremine-C) with BACE-1 (1W51) enzyme.

We investigated the molecular interaction between compound **I** (Bisacremine-C) and BACE-1 through Discovery Studio visualizer tools. We observed that the best-hit ligand (Bisacremine-C) and native ligand (non-peptidic inhibitor) occupied a similar catalytic site in the target protein (BACE-1) as shown in Figure 6A,B, where the reference inhibitor and BACE-1 complex has been stabilized by six conventional hydrogen bonds between THR72:HG1-LIG:O, GLN73:HN-LIG:O, THR232:HN-LIG:O, LIG:H-GLY230:O, LIG:H-ASP32:OD2, LIG:H-ASP228:OD2, one carbon–hydrogen bond between LIG:C-GLY11:O, one Pi-donor hydrogen bond between the THR231:HG1–LIG, two Pi-Pi T-shaped hydrophobic interactions between the TYR71–LIG, two alkyl hydrophobic interactions between the LIG-ILE110 and LIG:C-VAL69, and one Pi-alkyl hydrophobic interaction between TYR71-LIG:C. Moreover, several residues were also observed to make van der Walls interactions (Figure 6C). The Bisacremine-C (best hit) and BACE-1 complex has been stabilized with four conventional hydrogen bonds between the THR72:HN-Bisacremine-C:O and Bisacremine-C:H-GLY34:O. Moreover, several active site residues are involved in making van der Walls interactions between the complex (Figure 6D). Furthermore, we found that the Bisacremine-C bound with 9.04-fold higher affinity with BACE-1 than the native ligand. Due to its role in producing Aβ-42, a protein known for aggregating to the create Aβ plaque, increased beta-secretase (BACE-1) action may have negative consequences on the central nervous system (CNS). Polyphenols that regulate autophagy against neurodegeneration were able to reduce the toxicity of Aβ-42 and, ultimately, the aggregation of protein [72]. Consequently, reducing protein aggregation may be a more effective strategy for treating neurological diseases [73].

BACE-1 is, therefore, seen as a key target for preventing amyloid pathology and treating AD [16,74,75]. At a resolution of 2.55 Å, we employed the BACE-1 enzyme co-crystallized with hydroxyethyl amine inhibitor [48]. The catalytic function of the enzyme is mediated by the two aspartate residues (ASP32 and ASP228) [76,77]. Our findings demonstrated an interaction between Bisacremine-C and ASP228, a crucial catalytic residue. LEU30, GLY34, SER35, TYR71, THR72, GLN73, PHE108, ILE110, TRP115, ILE118, ILE126, TYR198, ILE226, ASP228, GLY230, THR232, and ARG235 are the common residues found in interaction of Bisacremine-C and reference ligand with BACE-1 protein. Our findings are consistent with the earlier study, which found that the BACE-1 inhibitors also interacted with GLY34, TYR71, and PHE108, in addition to ASP228 [78].

### 3.6. Molecular Dynamics Simulation

Between 1977 and 2002, 25 years, simulations based on the molecular dynamics of proteins saw rapid development and were used to solve a variety of issues [79]. Desmond, an application of software from Schrodinger LLC, was utilized to simulate molecular dynamics for 100 nanoseconds [44]. Docking experiments were the first stage of receptor and ligand complexes for molecular dynamics modeling. In static circumstances, the ligand-binding state can be predicted by molecular docking studies. Because docking offers a static image of a molecule’s binding posture at a protein’s active site, this is helpful [80]. However, molecular dynamics simulation generally simulates the atom motions for a time by incorporating Newton’s classical equation of motion by which the ligand interaction status in the physiological surroundings is anticipated [51,57]. 

#### 3.6.1. Analysis of Root-Mean-Square Deviation (RMSD) and RMSF

**Figure 7:**
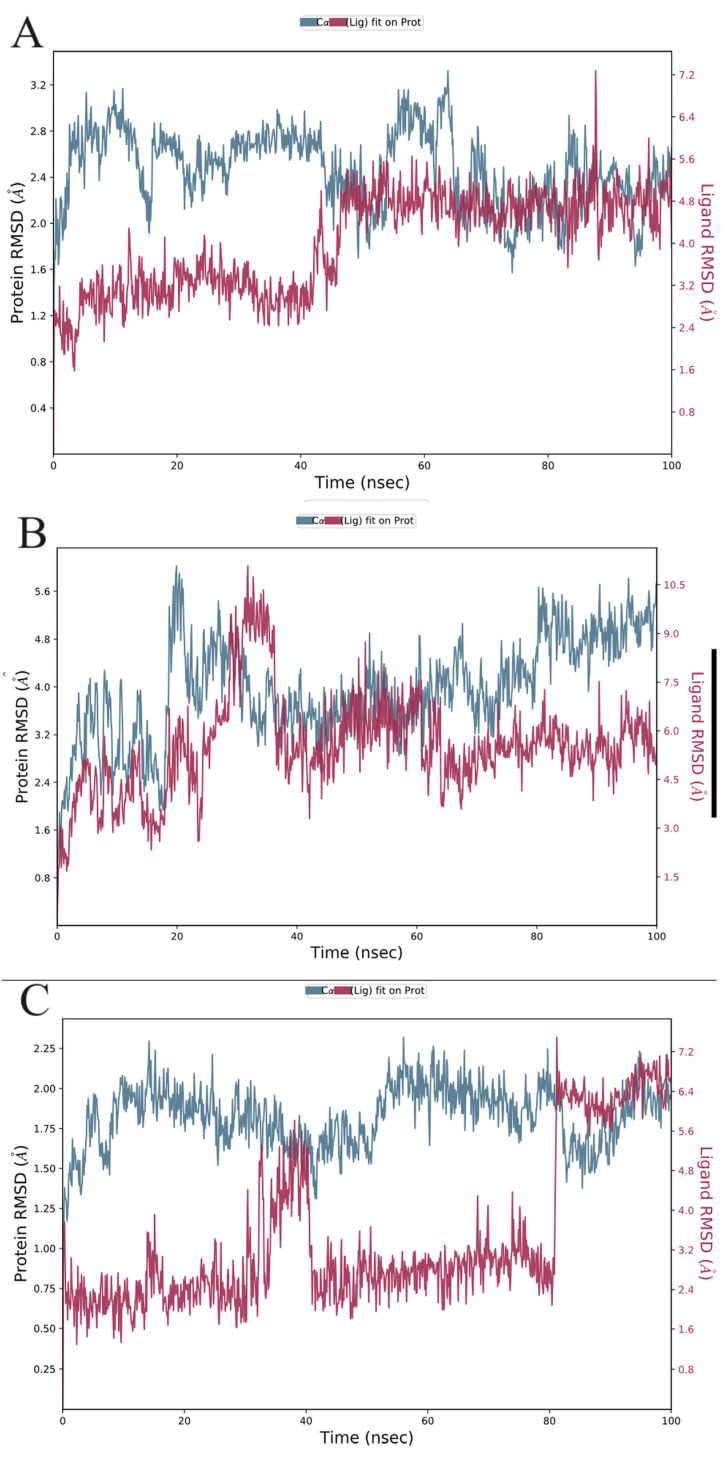
The C-alpha atoms of proteins and ligands that bind ((**A**): 1J1C-Bisacremine-C, (**B**): 1PBQ-Bisacremine-C, and (**C**): 1W51-Bisacremine-C) have been studied for their root-mean-square deviation (RMSD) over time. The protein RMSD’s temporal fluctuation is displayed on the left Y-axis. The ligand RMSD’s temporal fluctuation is displayed on the right Y-axis.

The measurement of RMSD offers an assessment of the stability and dynamic properties of the protein–ligand complex in molecular dynamics simulations. A protein or protein–ligand complex’s structural departure from its original posture has been assessed for the RMSD, which ultimately provides information on the stability of the protein–ligand complex throughout the simulation. Here, we describe how the RMSD of GSK-3β (1J1C), NMDA receptor (1PBQ), and BACE-1 (1W51) behave when simulated using molecular dynamics under physiological settings, either individually or in combination with Bisacremine-C (Figure 7). 

The progression of the RMSD values for the C-alpha atoms of ligand-bound proteins through time is shown in Figure 7. According to the RMSD plot (Figure 7A), the proteins in the complex 1J1C-Bisacremine-C attained stability at 20 ns. Following that time, RMSD variations are entirely acceptable and stay under 1.0 Angstrom up to 48 ns of the simulation. We noticed a flipping in ligand mode and regaining equilibrium at 55 ns. After that, the complex remained stable for the remainder of the simulation. 

Complex 1PBQ-Bisacremine-C’s RMSD plot (Figure 7B) demonstrates that the complex stabilized at 40 ns. The variation in RMSD values for protein after that stays within 1.5 Angstrom throughout the simulated duration. When ligands are fitted to proteins, the RMSD values fluctuate between 2.0 Angstrom and 100 ns. According to the RMSD plot, the proteins in the complex 1W51-Bisacremine-C (Figure 7C) attained stability at 5 ns. Following that, fluctuations in RMSD values remain within 1.0 Angstrom throughout the simulation duration. Until 38 ns, the RMSD values for the ligand fit to the protein varied within 1.0 Angstrom, but after that, there was a flip in the ligand mode, which attained equilibrium around 48 ns and then stayed stable until 82 ns. After switching to a different binding mode, it became stable again. This shows that the ligand stays persistently connected to the binding sites of each of the three targets during the simulation period. Our findings illustrated that the overall structures of target enzymes (1J1C, 1PBQ, and 1W51) did not change significantly due to the binding of Bisacremine-C, and the protein–ligand complexes remained perpetual throughout the simulation. During the initial period of simulation, we noticed the fluctuations in our protein–ligand complexes and, therefore, we performed an extended simulation of 50 ns of our protein–ligand complexes to check whether our complexes system achieved equilibrium even after 100 ns or not. We found that the system remains stable and achieves equilibrium during the extended simulation period (Supplementary Figure S2). Therefore, for the rest of the analysis, we focused on 100 ns of molecular dynamics simulation.

**Figure 8:**
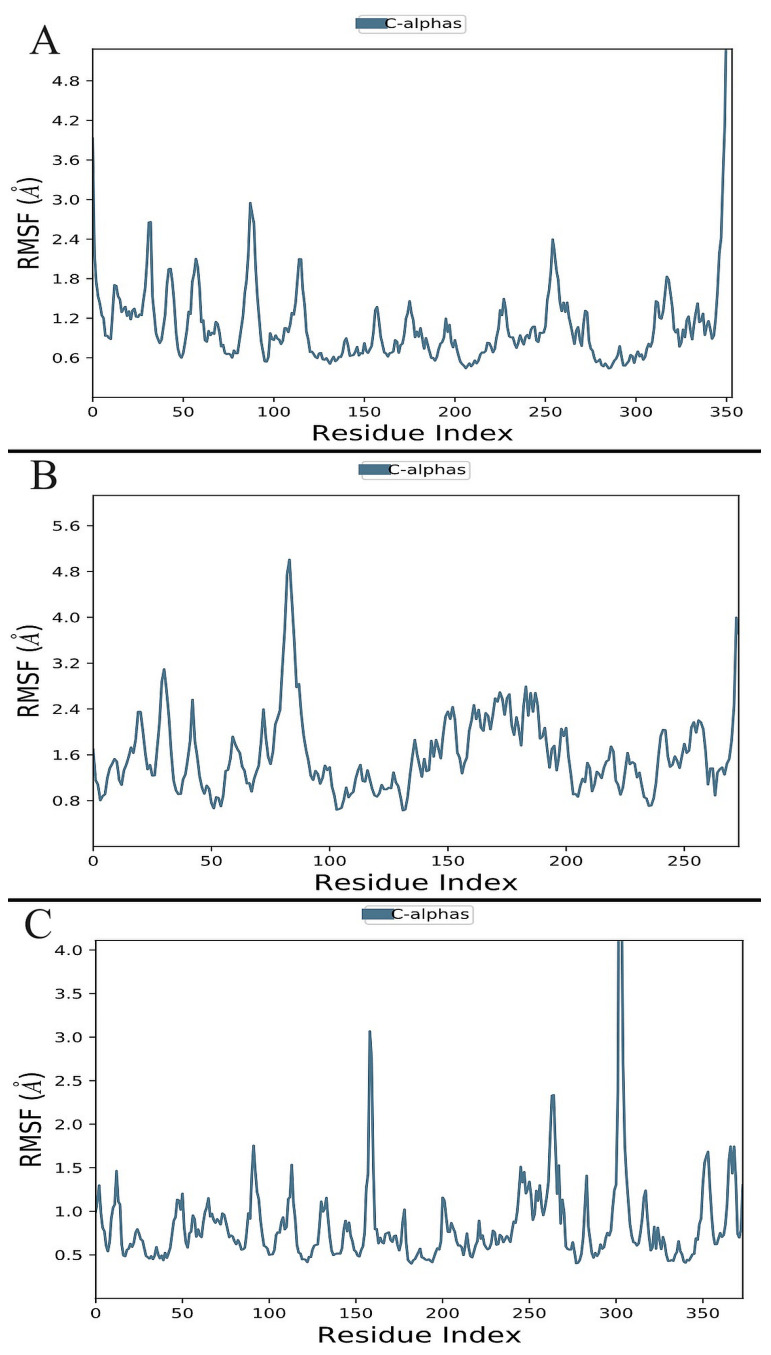
Residue-wise root-mean-square fluctuation (RMSF) of protein complexes ((**A**): 1J1C, (**B**): 1PBQ, (**C**): 1W51).

The RMSF values of the proteins that are bound to the ligand are shown in Figure 8 and, according to trajectories, illustrate that the residues with greater peaks are in loop regions or the N and C-terminal zones. Low RMSF values of the binding-site residues demonstrate the stability of ligand binding to the protein. In the presence of Bisacremine-C, the average RMSF values of BACE-1, GSK-3β, and NMDA receptor are 0.78 ± 0.06 Å, 1.61 ± 0.07 Å, and 0.95 ± 0.05 Å, respectively. According to these results, the protein–ligand combination is stable in nature, and the binding of the Bisacremine-C molecule did not significantly change the target protein’s overall structure.

#### 3.6.2. Secondary Structure Elements Analysis

**Figure 9:**
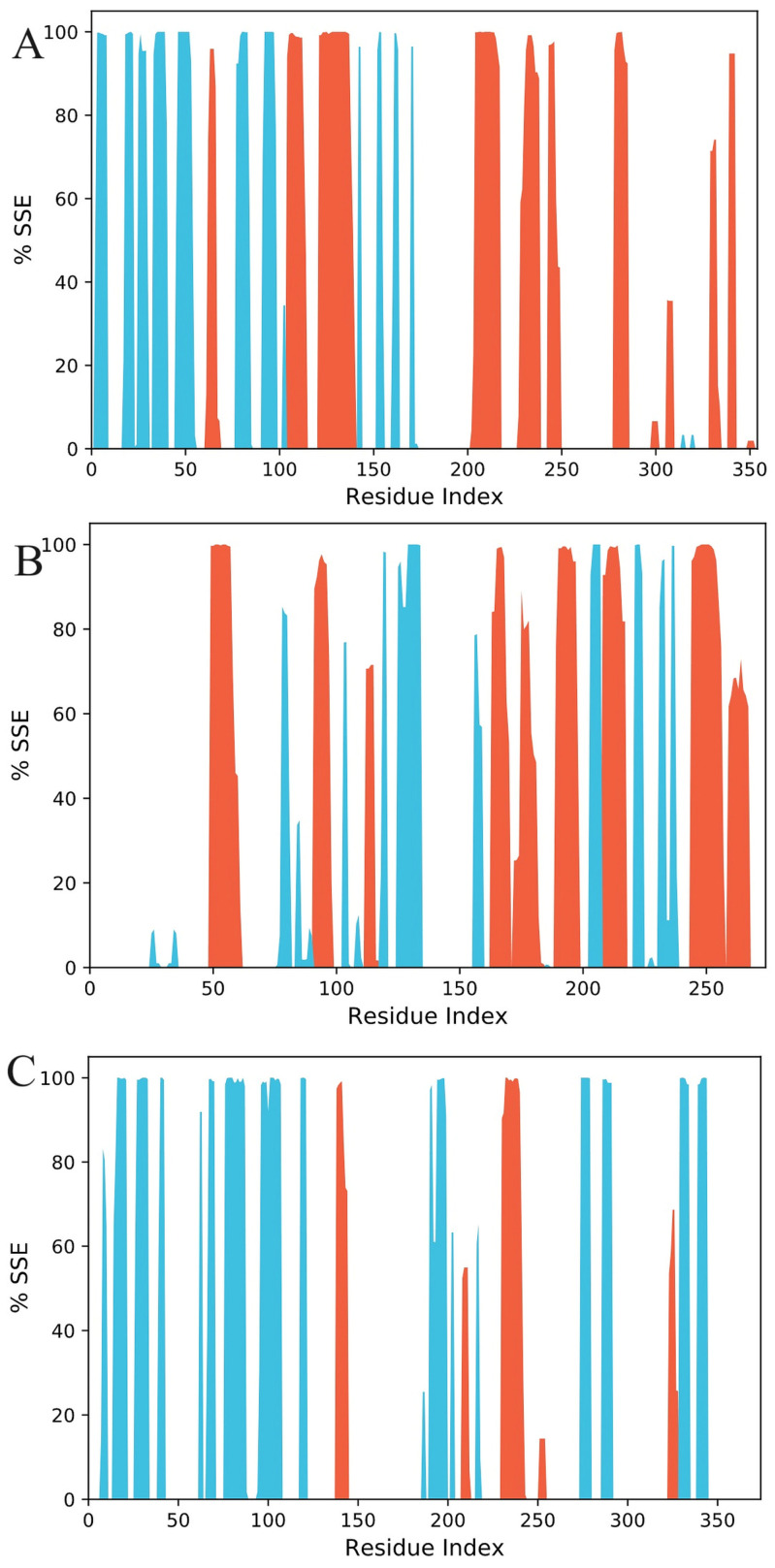
Distribution of the protein secondary structure elements by residue index in the complexed protein structures ((**A**): 1J1C, (**B**): 1PBQ, (**C**): 1W51). Red columns represent the alpha helix, and blue columns represent the beta strands.

Our results demonstrate that helix and strand were discovered to make up 20.3 and 16.9%, respectively, of 1J1C, while the secondary structure component was found to be 39.20%. For 1PBQ, the helix and strand percentages were 25.53% and 12.66%, respectively. Total SSE was 38.19%. The percentages of both helix and strand in the instance of 1W51 were 6.35% and 24.45%, respectively, and a total of 30.8% of secondary structural components were discovered (Figure 9).

#### 3.6.3. Histogram for Molecular Interactions of Protein–Ligand Complexes

**Figure 10:**
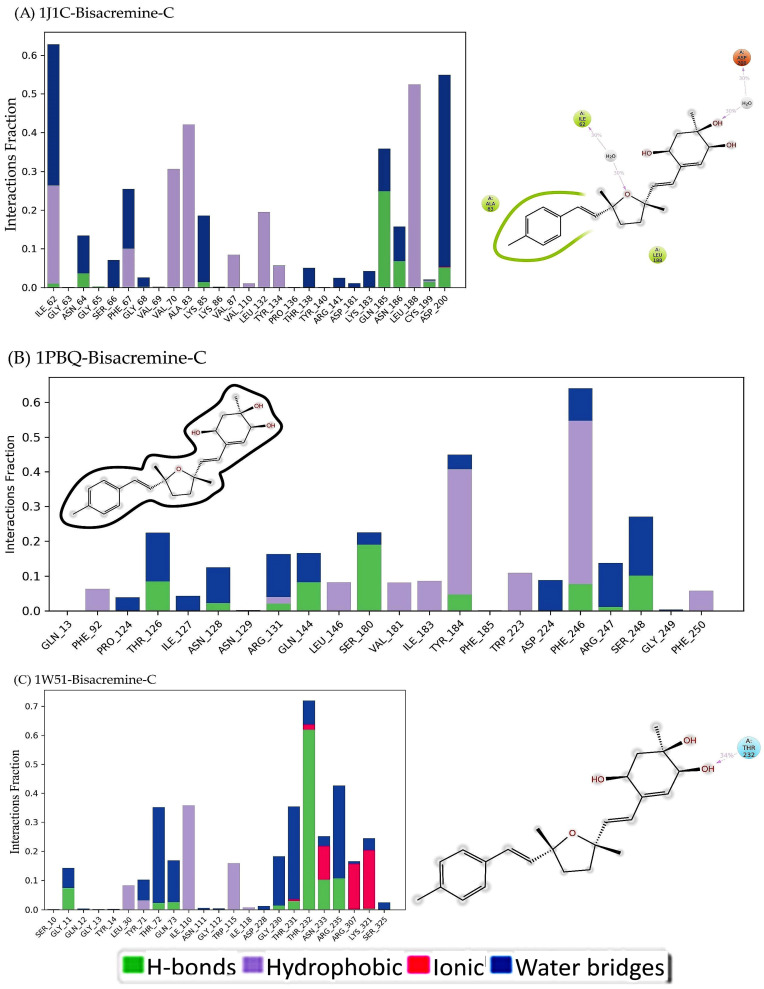
Histograms of protein–ligand complexes ((**A**): 1J1C-Bisacremine-C, (**B**): 1PBQ-Bisacremine-C, (**C**): 1W51-Bisacremine-C).

As can be observed in Figure 10, hydrogen bonds and hydrophobic interactions make up most of the notable ligand–protein interactions identified using MD simulation. The most significant hydrophobic contacts for the 1J1C-Bisacremine-C complex are with ILE62, VAL70, ALA83, and LEU188, whereas GLN 185 is significant for H-bonds (Figure 10A). In terms of hydrophobic contacts, TYR184 and PHE 246 are the most important, while SER180 is vital for H-bonds in 1PBQ-Bisacremine-C (Figure 10B). THR232 is most crucial as H-bonds for 1W51-Bisacremine-C and ILE110 produced hydrophobic contacts (Figure 10C). In these bar charts of the histogram, a value of 1.0 signifies over the trajectory that the connections remained intact for 100% of the simulation period. We notice that the compound **I** (Bisacremine-C) has a two-methyl group in the tetrahydrofuran ring that is in cis-configuration similar to the configuration of the hydroxyl group. Hence, fewer steric hindrances were observed. Therefore, it has better binding interactions with target proteins.

#### 3.6.4. Analysis of Solvent-Accessible Surface Area (SASA) and Radius of Gyration (Rg)

**Figure 11:**
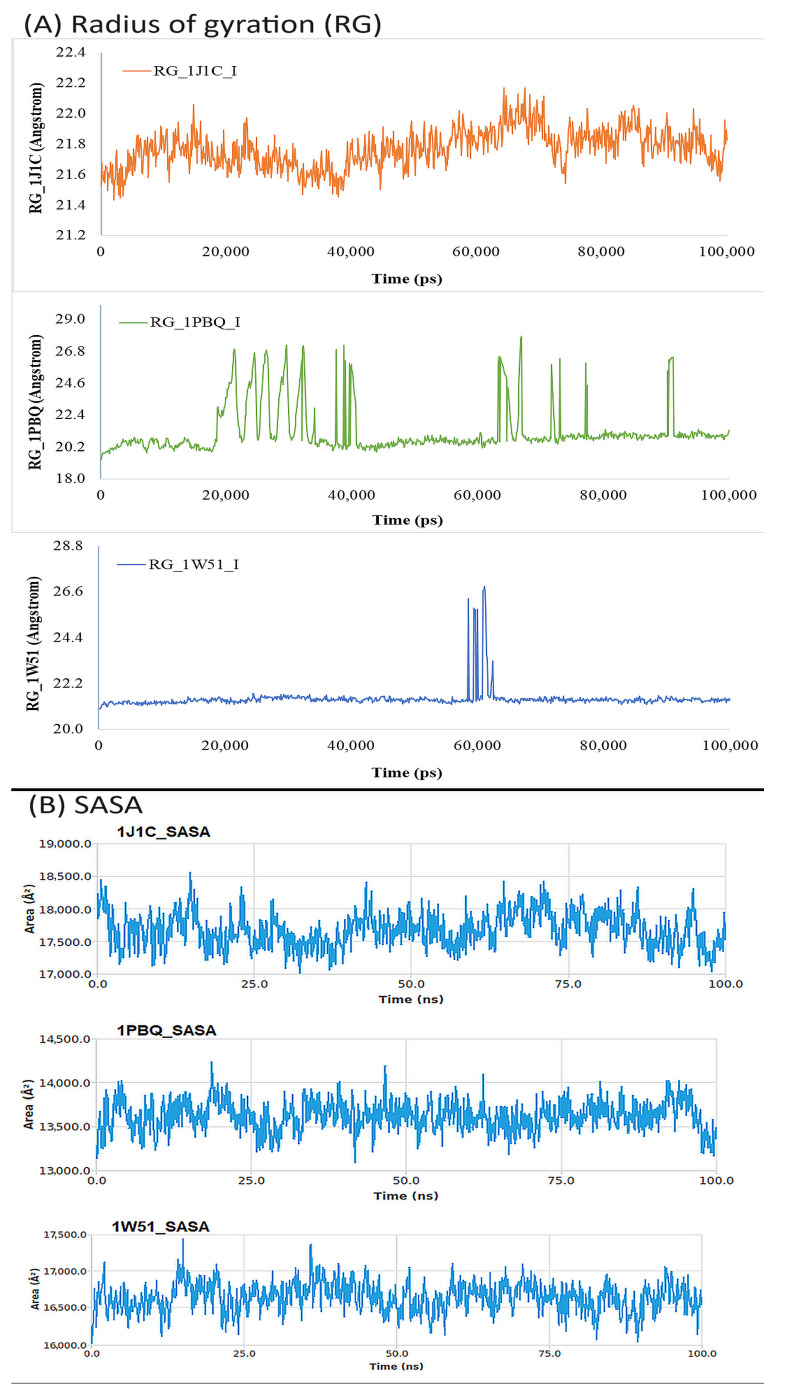
(**A**) The radius of gyration and (**B**) SASA calculated for three target proteins (1J1C, 1PBQ, and 1W51) bound with compound I (Bisacremine-C).

The relationship between a ligand’s radius of gyration (Rg) as well as solvent-accessible surface area (SASA) in relation to simulation time reveals details about the ligand’s behavior inside the enzyme’s binding pocket [81]. The radius of gyration (Rg) refers to the arrangement of atoms in a protein’s structure around its axis. The distance that exists between the spinning point and the location where the energy exchange has the greatest impact is represented by the length Rg. The identification of various polymer types, including proteins, is made easier with the use of this conceptual notion. Calculating Rg and measuring distance are the two key indicators for predicting the structural activity of a macromolecule. A conformational shift occurs when a ligand/lead molecule attaches to a protein, which alters the radius of gyration. A sophisticated computer method for calculating the radius of gyration may be used to track a protein’s compactness, which is directly connected to the rate of folding of a protein. Figure 11A shows the fluctuation in Rg of Bisacremine-C coupled to several proteins (1J1C, 1PBQ, and 1W51) as a function of simulation duration. The findings demonstrate that during the simulation, the Rg values of several protein–ligand complexes varied within the permissible range. The average Rg values of 1J1C, 1PBQ, and 1W51 bound with Bisacremine-C were estimated as 21.77 ± 0.13 Å, 21.27 ± 1.63 Å, and 21.47 ± 0.53 Å, respectively.

When a ligand binds to a protein, the solvent-accessible surface area (SASA), which measures the protein’s exposure to the solvent, may determine if the protein is in its native form. Here, the SASA of the target proteins 1J1C, 1PBQ, and 1W51 bound to Bisacremine-C was evaluated (Figure 11B). The SASA of complexes varied slightly within the acceptable limits. The average SASA values of Bisacremine-C bound with 1J1C, 1PBQ, and 1W51 were 17,680.6 ± 261.9 Å^2^, 13,620.2 ± 168.2 Å^2^, and 16,638.9 ± 194.8 Å^2^, respectively. These results suggest that Bisacremine-C remained inside the binding cavity of 1J1C, 1PBQ, and 1W51 in a stable conformation.

### 3.7. Calculations of Prime-MM/GBSA (Free Energy)

**Figure 12:**
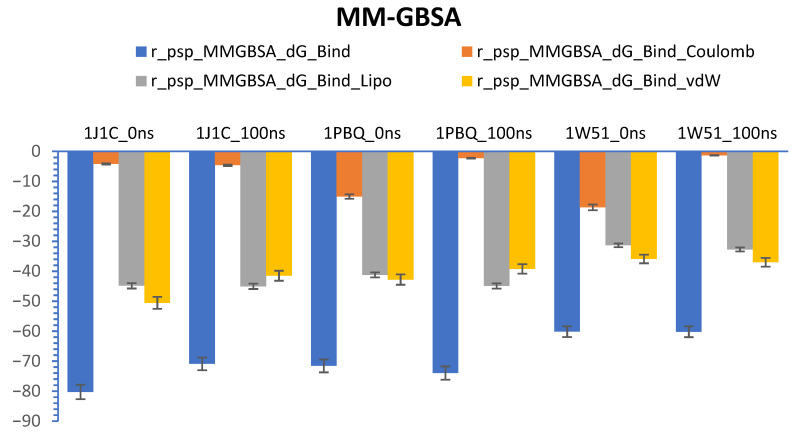
Graphs show the values of MM-GBSA estimated at 0 and 100 ns of simulation time.

Prime-MM/GBSA (free energy) computation is known as a reliable technique for determining the equilibrium of protein–ligand complex in a solvent system [82]. We represented the calculation of delta G (dG) through prime-MM/GBSA of target proteins (1J1C, 1PBQ, and 1W51) and ligand (Bisacremine-C) complex at 0 ns (starting time of computation) and 100 ns (end time of computation) in Figure 12. Our results demonstrate that the dG average value for the 1PBQ-Bisacremine-C complex is −72.7680 ± 1.19, with a range between −739,604 and −71,576. The dG average value for the 1J1C-Bisacremine-C complex is −75.5606 ± 4.69, with a range between −80.2499 and −70.8712. Moreover, the dG average value for the 1W51-Bisacremine-C complex is –60.1663 ± 0.01, with a range between −60.1801 and −60.1525. We notice that van der Waals energy (GvdW) and lipophilic energy (GSol_Lipo) or non-polar solvation influence the development of the protein–ligand complex in a stable form.

## 4. Conclusions

In this study, we theoretically investigated entire fungal metabolites available on the PubChem database for the management of Alzheimer’s disease by targeting three key regulatory proteins of three different pathophysiological pathways in the progression of AD. Our multi-targeted approach concluded that compound **I** (Bisacremine-C), as shown in Figure 3, is the most promising fungal metabolite, having a 25-fold higher affinity with GSK-3β, 6.3-fold higher affinity with NMDA, and 9.04-fold higher affinity with BACE-1 than their native ligands, respectively. This compound is also found to be safer and non-toxic, even at a higher dose of 5000 mg/kg BW. The molecular dynamics simulation validated that the overall structures of targeted enzymes did not change significantly after the binding of Bisacremine-C, and the ligand remained inside the binding cavity in a stable conformation for most of the simulation time. This study laid a foundation for further experimental validation and clinical trials for the biopotency of Bisacremine-C.

## Figures and Tables

**Table 1:** Physicochemical and pharmacokinetic criteria of fungal metabolites.

Compounds Code	PubChem CID		MW	#HA	#AHA	F-Csp3	#RB	#HBA	#HBD	MR	TPSA	XLOGP3
**A**	22216483	R-N-DMAT	272.34	20	9	0.31	5	3	2	80.9	68.25	0
**B**	46216805	Daedalin A	192.21	14	6	0.27	1	3	2	53.75	49.69	1.44
**C**	46880982	See C in footer	233.26	17	9	0.31	5	3	2	64.94	62.32	2.28
**D**	60166720	Diaportheone B	220.22	16	6	0.42	0	4	2	56.51	66.76	1.54
**E**	122187709	Bisacremine A	384.51	28	6	0.5	4	4	3	113.11	69.92	2.36
**F**	122206138	Baccinol H	290.35	21	6	0.47	5	4	2	81.68	66.76	2.21
**G**	139583580	See G in footer	251.11	15	6	0.4	3	3	2	59.86	49.69	2.05
**H**	139586224	Bisacremine D	384.51	28	6	0.5	4	4	3	113.11	69.92	2.36
**I**	139587420	Bisacremine-C	384.51	28	6	0.5	4	4	3	113.11	69.92	2.36
**J**	139587958	Bisacremine B	384.51	28	6	0.5	4	4	3	113.11	69.92	2.36
**K**	139588462	Penipaline B	312.41	23	9	0.42	3	3	3	97.44	65.12	1.35
**L**	139589365	Emefuran D	262.3	19	6	0.4	4	4	2	72.47	66.76	2.25
**M**	139591664	See M in footer	228.63	15	6	0.3	2	4	2	53.76	66.76	0.95
**N**	145720807	Phexandiol B	208.25	15	6	0.5	3	3	2	57.34	49.69	2.07

Note: MW—molecular weight; #HA—number of hetero atoms; #AHA—number of aromatic heteroatoms; #RB—number of rotatable bonds; #HBA—number of hydrogen bond acceptor; #HBD—number of hydrogen bond donors; MR—molar refractivity; TPSA—total plasmon surface area; XLOGP3—partition coefficient; **C**—2-Hydroxypropanoic acid 2-(1H-indole-3-yl)ethyl ester; **G**—Erythro-1-(3′,5′-dichloro-4′-methoxyphenyl)-1,2-propanediol; **M**—2-[(1S)-5-Chloro-4-hydroxy-1,3-dihydro-2-benzofuran-1-yl]acetic acid.

**Table 2:** Prediction of toxicity level of fungal metabolites.

Code	Hepatotoxicity (Probability)	Carcinogenicity (Probability)	Immunotoxicity (Probability)	Mutagenicity (Probability)	Cytotoxicity (Probability)	Predicted LD_50_ (mg/kg)
**A**	IA (0.68)	IA (0.70)	IA (0.99)	IA (0.74)	IA (0.79)	225
**B**	IA (0.80)	IA (0.59)	Active (0.75)	IA (0.64)	IA (0.69)	500
**C**	IA (0.57)	IA (0.66)	IA (0.96)	IA (0.77)	IA (0.80)	3500
**D**	IA (0.73)	IA (0.55)	IA (0.66)	IA (0.62)	IA (0.66)	1060
**E**	IA (0.78)	IA (0.56)	IA (0.69)	IA (0.72)	IA (0.75)	5000
**F**	IA (0.83)	IA (0.57)	Active (0.83)	IA (0.61)	IA (0.76)	1500
**G**	IA (0.73)	IA (0.57)	IA (0.69)	IA (0.75)	IA (0.68)	1040
**H**	IA (0.78)	IA (0.56)	IA (0.69)	IA (0.72)	IA (0.75)	5000
**I**	IA (0.78)	IA (0.56)	IA (0.69)	IA (0.72)	IA (0.75)	5000
**J**	IA (0.78)	IA (0.56)	IA (0.69)	IA (0.72)	IA (0.75)	5000
**K**	IA (0.63)	IA (0.70)	IA (0.86)	IA (0.72)	IA (0.70)	550
**L**	IA (0.79)	IA (0.60)	Active (0.69)	IA (0.61)	IA (0.77)	1000
**M**	IA (0.66)	IA (0.61)	IA (0.97)	IA (0.70)	IA (0.68)	1500
**N**	IA (0.71)	IA (0.68)	IA (0.96)	IA (0.62)	IA (0.81)	1295

Inactive: IA; In brackets, the probability of activeness or inactiveness is mentioned on a 0–1 scale where 0 means no (0%) chance of concerned property and 1 means 100% chance of concerned property.

**Table 3:** Binding energy and affinity values through molecular docking of fungal metabolites.

	Binding Energy (ΔG: Kcal/mol)			Binding Affinity (Ki: M^−1^)		
Code	GSK-3β (1J1C)	NMDA (1PBQ)	BACE-1 (1W51)	GSK-3β (1J1C)	NMDA (1PBQ)	BACE-1 (1W51)
**A**	−7.4 ± 0.2	−7.2 ± 0.1	−7.1 ± 0.1	2.7 × 10^5^	1.9 × 10^5^	1.6 × 10^5^
**B**	−6 ± 0.3	−6.3 ± 0.1	−6.5 ± 0.2	2.5 × 10^4^	4.1 × 10^4^	5.8 × 10^4^
**C**	−6.3 ± 0.1	−7 ± 0.3	−6.7 ± 0.1	4.1 × 10^4^	1.4 × 10^5^	8.2 × 10^4^
**D**	−7.2 ± 0.1	−7.5 ± 0.1	−6.8 ± 0.1	1.9 × 10^5^	3.1 × 10^5^	9.6 × 10^4^
**E**	−8.2 ± 0.2	−8.6 ± 0.3	−8.2 ± 0.2	1.0 × 10^6^	2.0 × 10^6^	1.0 × 10^6^
**F**	−7 ± 0.1	−7.6 ± 0.1	−7.6 ± 0.1	1.4 × 10^5^	3.7 × 10^5^	3.7 × 10^5^
**G**	−5.8 ± 0.1	−5.5 ± 0.1	−5.7 ± 0.1	1.8 × 10^4^	1.1 × 10^4^	1.5 × 10^4^
**H**	−8.6 ± 0.2	−9.5 ± 0.2	−9.3 ± 0.1	2.0 × 10^6^	9.2 × 10^6^	6.6 × 10^6^
**I**	−8.7 ± 0.2	−9.5 ± 0.1	−9.1 ± 0.2	2.4 × 10^6^	9.2 × 10^6^	4.7 × 10^6^
**J**	−7.6 ± 0.1	−8.9 ± 0.2	−9.3 ± 0.2	3.7 × 10^5^	3.3 × 10^6^	6.6 × 10^6^
**K**	−8 ± 0.1	−8.5 ± 0.2	−8.4 ± 0.1	7.3 × 10^5^	1.7 × 10^6^	1.4 × 10^6^
**L**	−6.9 ± 0.1	−7.9 ± 0.2	−6.9 ± 0.1	1.1 × 10^5^	6.2 × 10^5^	1.1 × 10^5^
**M**	−6.1 ± 0.1	−7.1 ± 0.1	−6.1 ± 0.1	2.9 × 10^4^	1.6 × 10^5^	2.9 × 10^4^
**N**	−6.3 ± 0.1	−6.7 ± 0.1	−6.6 ± 0.2	4.1 × 10^4^	8.1 × 10^4^	6.9 × 10^4^
**NL**	−6.8 ± 0.1	−8.4 ± 0.2	−7.8 ± 0.1	9.6 × 10^4^	1.4 × 10^6^	5.2 × 10^5^

## Data Availability

All data generated or analyzed during this study are included in the published article.

## Supplementary Materials

The following supporting information can be downloaded at: https://www.mdpi.com/article/10.3390/biom13111613/s1, Supplementary Figure S1. 2D structure of native ligands and best hit (Bisacremine-C), Supplementary Figure S2. The C-alpha atoms of proteins and ligands that bind (A: 1J1C-Bisacremine-C, B: 1PBQ-Bisacremine-C, and C: 1W51-Bisacremine-C) have been studied for their root-mean-square deviation (RMSD) over time of 0–150 ns.
