# Supplementary material for: Pharmacophore-Based Screening, Molecular Docking, and Dynamic Simulation of Fungal Metabolites as Inhibitors of Multi-Targets in Neurodegenerative Disorders
Source: Biomolecules. 2023 Nov 4;13(11):1613. doi: 10.3390/biom13111613 (PMC10669353; doi:10.3390/biom13111613)
Supplement: Supplementary file 1 [file biomolecules-13-01613-s001.zip › Supplementary information-2651855.pdf]

Supplementary information

Pharmacophore-Based Screening, Molecular Docking, and Dynamic Simulation of Fungal Metabolites as Inhibitors of Multi-Targets in Neurodegenerative Disorders

Danish Iqbal [1,*,†], Mohammed Alsaweed [2], Qazi Mohammad Sajid Jamal [3], Mohammad Rehan Asad [4], Syed Mohd Danish Rizvi [5], Moattar Raza Rizvi [6], Hind Muteb Albadrani [7], Munerah Hamed [8], Sadaf Jahan [2] and Hadeel Alyenbaawi [2,*,†]

[1] Department of Health Information Management, College of Applied Medical Sciences, Buraydah Private Colleges, Buraydah 51418, Saudi Arabia
[2] Department of Medical Laboratory Sciences, College of Applied Medical Sciences, Majmaah University, Majmaah 11952, Saudi Arabia; m.alsaweed@mu.edu.sa (M.A.); s.jahan@mu.edu.sa (S.J.)
[3] Department of Health Informatics, College of Public Health and Health Informatics, Qassim University, Al Bukayriyah 52741, Saudi Arabia; m.quazi@qu.edu.sa
[4] Department of Basic Medical Science, College of Medicine, Majmaah University, Al Majmaah 11952, Saudi Arabia; mr.asad@mu.edu.sa
[5] Department of Pharmaceutics, College of Pharmacy, University of Ha'il, Ha'il 81442, Saudi Arabia; sm.danish@uoh.edu.sa
[6] School of Allied Health Sciences, Manav Rachna International Institute of Research & Studies (MRIIRS), Faridabad 121001, India; rajrizvi@gmail.com
[7] Department of Clinical Laboratory Sciences, College of Applied Medical Sciences, Imam Abdulrahman Bin Faisal University, Dammam 34212, Saudi Arabia; hmalbadrani@iau.edu.sa
[8] Department of Pathology, Faculty of Medicine, Umm Al-Qura University, Makkah 21955, Saudi Arabia; mhhamed@uqu.edu.sa
* Correspondence: danishiqbalmail@gmail.com or danish.khan@bpc.edu.sa (D.I.); hadeel.a@mu.edu.sa (H.A.)
† These authors contributed equally to this work.

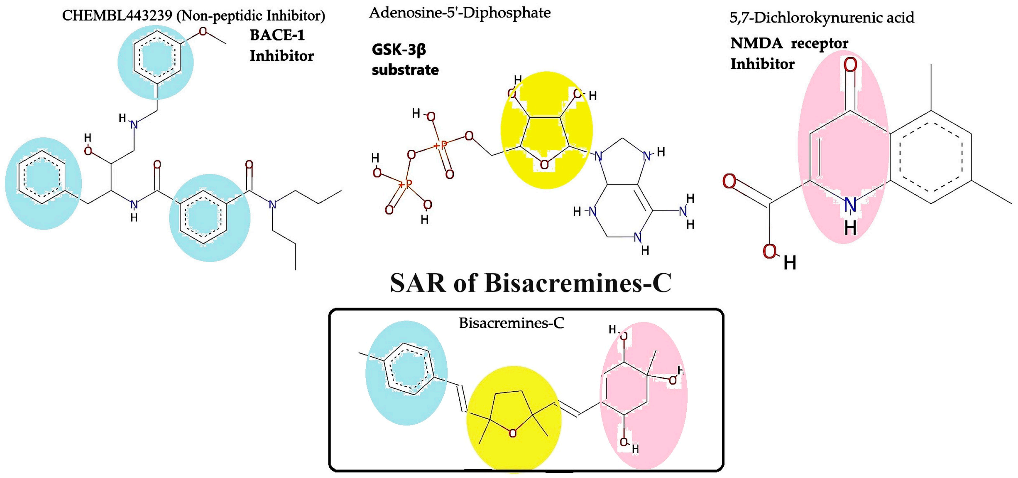

Supplimentary Figure S1. Structure activity relationship of Bisacremine-C with the native ligands of respective proteins.

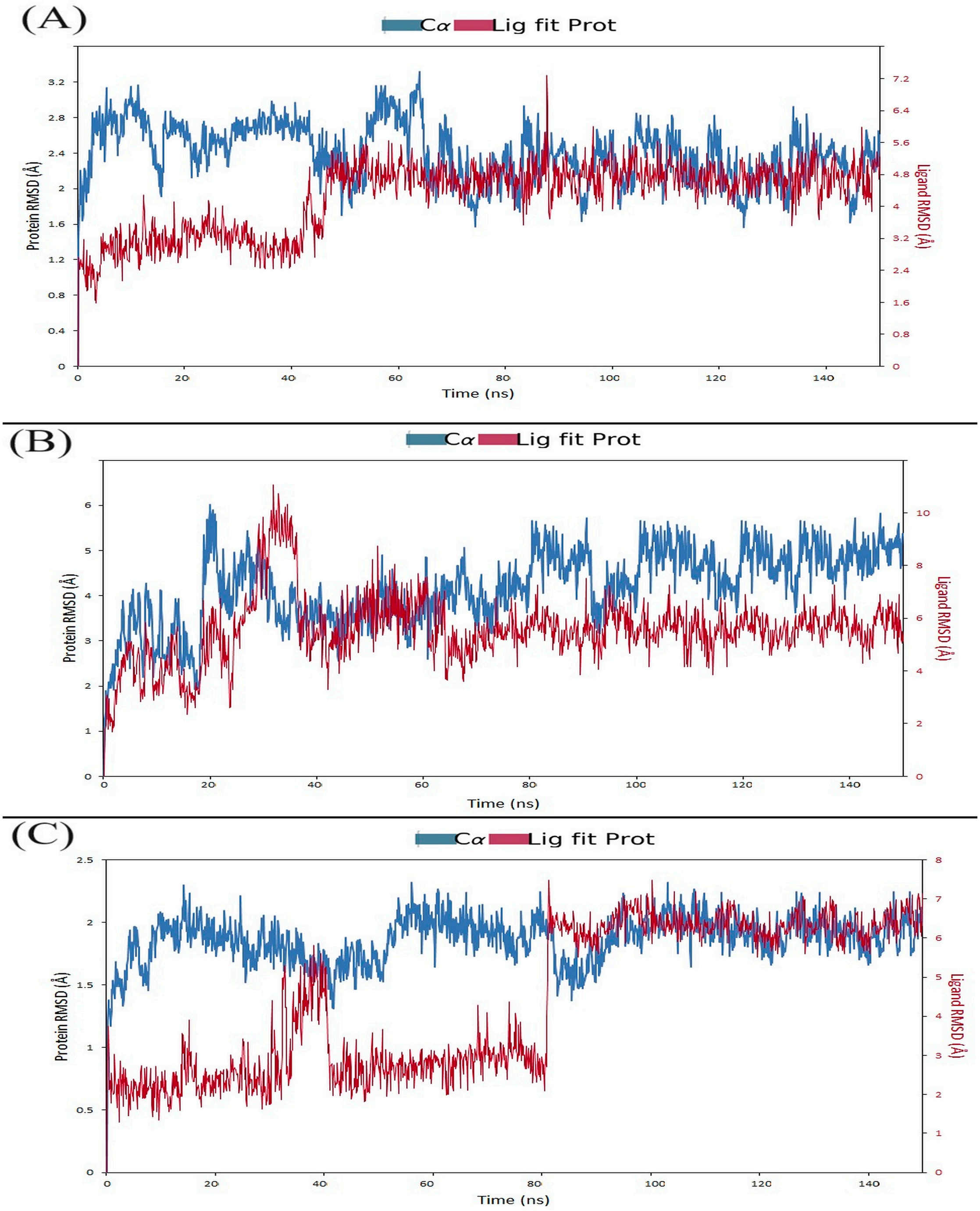

Supplementary Figure S2. The C-alpha atoms of proteins and ligands that bind (A: 1J1C-Bisacremine-C, B: 1PBQ-Bisacremine-C, and C: 1W51- Bisacremine-C) have been studied for their root mean square deviation (RMSD) over time of 0-150 ns. The protein RMSD's temporal fluctuation is displayed on the left Y-axis. The ligand RMSD's temporal fluctuation is displayed on the right Y-axis.
